# Cytological analysis of flower development, insights into suitable growth area and genomic background: implications for *Glehnia littoralis* conservation and sustainable utilization

**DOI:** 10.1186/s12870-024-05585-5

**Published:** 2024-09-30

**Authors:** Chang An, Kangzhuo Ye, Runfa Jiang, Jiayi Chen, Yixin Yao, Lin Lu, Yan Cheng, Ruoyu Liu, Xiaofen Liu, Heming Zhao, Yuan Qin, Ping Zheng

**Affiliations:** 1 Fujian Provincial Key laboratory of Haixia applied plant systems biology, Haixia Institute of Science and Technology and College of Life Sciences, Fujian Agriculture and Forestry University, Fuzhou, 350002 China; 2 Fujian Key Laboratory of Island Monitoring and Ecological Development (Island Research Center, MNR), Fuzhou, 350002 China; 3 Macau Centre for Research and Development in Chinese Medicine, State Key Laboratory of Quality Research in Chinese Medicine, Institute of Chinese Medical Sciences, University of Macau, Macao, China; 4 College of Pharmacy, Fujian University of Traditional Chinese Medicine, Fuzhou, 350122 China; 5 Pingtan Science and Technology Research Institute, College of marine sciences, Fujian Agriculture and Forestry University, Fuzhou, 350002 China

**Keywords:** *Glehnia littoralis*, Flower development, Suitable growth area, Genomic background

## Abstract

**Background:**

*Glehnia littoralis* F. Schmidt ex Miq., an endangered plant species with significant medicinal, edible, and ecological value, is now a central concern for conservation and sustainable utilization. Investigating the physiological and ecological mechanisms leading to its endangerment and elucidating its genetic background constitutes the foundation for conducting in-depth research on *G. littoralis*.

**Results:**

Our observations have revealed a significant degree of floral sterility in wild populations of *G. littoralis*. The inflorescences of *G. littoralis* are classified into three types: completely fertile, completely sterile, and partially fertile compound umbels. Moreover, the flowers of *G. littoralis* can be categorized into fertile and sterile types. Sterile flowers exhibited abnormalities in the stigma, ovary, and ovules. This study is the first to discover that the presence or absence of a giant cell at the funiculus during the initiation of ovule primordium determines whether the flower can develop normally, providing cytological evidence for female sterility in *G. littoralis*. Conversely, both fertile and sterile flowers produced normally developed pollen. Field observations have suggested that robust plants bear more fertile umbels, while weaker ones have fewer or even no fertile umbels, indicating a close relationship between flower fertility and plant nutritional status. Our model correctly predicted that the eastern coastal regions of China, as well as prospective areas in Neimenggu and Sichuan, are suitable environments for its cultivation. Additionally, Using flow cytometry and genome survey, we estimated the genome size of *G. littoralis* to be 3.06 Gb and the heterozygosity to be 4.58%.

**Conclusion:**

The observations and findings presented in this study were expected to provide valuable insights for further conserving its genetic resources and sustainable utilization of *G. littoralis*.

**Supplementary Information:**

The online version contains supplementary material available at 10.1186/s12870-024-05585-5.

## Background

*Glehnia littoralis*, a coastal endemic perennial herb belonging to the Apiaceae family [1, 2], has been extensively documented for its medicinal properties across traditional Chinese, Japanese, and Korean medicine, where its dried roots are highly valued for their yin-nourishing, lung-moistening, stomach-strengthening, and fluid-generating properties. Phytochemical studies have demonstrated that the roots of *G. littoralis* are rich in polyphenolic compounds and furanocoumarins, which exhibit potent antioxidant, anti-inflammatory, antitumor, and immunomodulatory properties [3–5]. Currently, it is an integral component in numerous traditional medicinal formulations aimed at enhancing immune function and treating various respiratory disorders [6]. Furthermore, the roots and tender stems and leaves of *G. littoralis* are edible and have garnered widespread popularity as a common vegetable and health food, particularly in Japan, the United States, and Southeast Asia [7–9].

The wild populations of *G. littoralis*, a keystone species inhabiting the temperate sandy coastlines of the North Pacific, play a critical ecological role within coastal ecosystems [10]. Unfortunately, due to habitat loss and unsustainable exploitation, these wild populations are now under imminent threat of extinction [11]. Compounding this issue is the species’ low seed germination rate, which presents challenges for artificial propagation and cultivation [12]. Current research suggests that nutritional limitations, poor pollination and fertilization, and adverse weather conditions are likely causes of seed abortion in *G. littoralis*. Studies have also demonstrated that *G. littoralis* exhibits strong apical dominance during its flowering and fruiting phases, where the central, terminal flowers and fruits of the compound umbels on the same plant tend to develop preferentially. Consequently, flower and fruit abortion is commonly observed during the transition from flowering to fruit maturity. The insufficient nutrient supply often leads to incomplete development of the embryo and endosperm, resulting in shrunken or empty seeds [13]. Continuous observation of flower development in *G. littoralis* has also revealed instances of abortion and defects in pistil or ovule development, with abnormalities in embryonic and endosperm development being noted [14, 15]. Clearly, many aspects of the reproductive development of *G. littoralis* remain unclear, such as the cytological basis of apical dominance and the relationship between reproductive defects and nutritional limitations. Detailed observation and further research are necessary to address these issues. Therefore, the study of the biology, particularly the reproductive biology, of *G. littoralis* is crucial for understanding its endangerment mechanisms and for laying the foundation for its conservation and sustainable utilization.

One noteworthy emerging conservation strategy gaining traction in the realm of plant conservation and protected area management is the reintroduction of plant species into their natural or semi-natural habitats [16, 17]. However, the long-term viability of such reintroduction efforts is contingent upon a comprehensive assessment of the dynamic context of climatic and other environmental changes [18]. Due to its recognized therapeutic efficacy, *G. littoralis* has been transplanted for cultivation in inland fields [19, 20]. As a species with a narrow distribution, concerns have been raised about whether ex situ cultivation can provide optimal conditions for its survival. Predicting the suitable growth regions for *G. littoralis* can provide foundational insights crucial for conservation strategies, targeted breeding initiatives, and sustainable management practices in both agricultural and natural habitats. Furthermore, the preservation and utilization of the rich genetic diversity of *G. littoralis* are intricately linked to the quality of its seedlings, which is crucial for realizing its medicinal and nutritional potential [21–23]. In recent years, advances in molecular biology have led to the sequencing of many plant genomes [24–27]; however, the genomic background of *G. littoralis* remains insufficiently explored. Conducting genomic sequencing for this species could offer crucial insights for the conservation and breeding of its genetic diversity and population resources.

In light of the aforementioned context, this study utilized a combination of inflorescence microscopy and fluorescence microscopy to comprehensively observe the whole-process of reproductive development and floral morphology of *G. littoralis*. We provided a detailed elucidation of the developmental characteristics of its inflorescences and described the process of male and female gametophyte development. For the first time, we discovered that the presence of a giant cell at the funiculus during the initiation of the ovule primordium plays a critical role in normal flower development, thereby clarifying the cytological basis of apical dominance in *G. littoralis*. Additionally, we utilized the maximum-entropy (MaxEnt) model to predict the suitable growth regions for *G. littoralis* in China. Finally, using flow cytometry and genomic analysis, we estimated its genome size and heterozygosity. These findings will enhance our understanding of *G. littoralis* and establish a foundation for future research, conservation, and utilization of this endangered species.

## Materials and methods

### Observation of umbels morphology, male gametophyte and female gametophyte

The field investigation and sample collection for this study focused on a wild population of *G. littoralis* located at Changjiang ‘ao Beach, Pingtan County, Fujian Province, China (N 37° 28′ 27″, E 121° 27′ 29″), identified by Professor Chengzi Yang from Fujian University of Traditional Chinese Medicine. The voucher specimen (Accession no: 35012822043001LY) is deposited in the Herbarium of Fujian University of Traditional Chinese Medicine (FJTCM**)**. During April and May, corresponding with the flowering period of *G. littoralis*, we conducted fieldwork at Changjiang Ao Beach and documented the inflorescence characteristics at various developmental stages using a Nikon D7200 digital camera. Then, we collected the flowers of *G. littoralis* at different developmental stages without damaging the whole inflorescence and brought them back to the laboratory in an ice box for further observation. Flower buds at different developmental stages were dissected under a stereoscope using forceps and dissecting needles. They were then placed on 0.8% agar plates for photographs, and photographs were taken using a Leica DFC550 microscope and measured using ImageJ software (NIH).

### Observation of umbels morphology, male gametophyte and female gametophyte

Further, we went to Changjiang Ao Beach during the flowering season to conduct field observations, and recorded the characteristics of different stages of inflorescences using a Nikon D7200 digital camera. Then, we collected the flowers of *G. littoralis* at different developmental stages without damaging the whole inflorescence, and brought them back to the laboratory in an ice box for further observation. Flower buds at different developmental stages were dissected under a stereoscope using forceps and dissecting needles. They were then placed on 0.8% agar plates for photographs, and photographs were taken using a Leica DFC550 microscope and measured using ImageJ software (NIH).

Male gametophyte development was assessed through a combination of differential interference contrast (DIC) microscopy and inflorescence microscopy. In the case of DIC microscopy, pollen grains at various developmental stages were collected and subjected to clearing using a chloral hydrate solution (comprising chloral hydrate, H2O, and glycerol in an 8:2:1 ratio), and subsequently mounted on glass slides. Cleared anthers were visualized employing a BX63 microscope (Olympus) equipped with DIC optics. In the context of inflorescence microscopy, the specimens underwent a decolorization process using a 25% acetic acid and 75% ethanol solution, which was repeated thrice. Subsequently, they were stained with 4’, 6-Diamidino-2-Phenylindole (DAPI), following the established protocol as outlined by previous studies [28, 29]. The male gametophyte nuclei were then examined utilizing Leica MZ10F and DM2500 microscopes.

The flower buds at various developmental stages were harvested and subjected to fixation in FAA solution (comprising 50% ethanol, glacial acetic acid, and formaldehyde in an 89:6:5 ratio) for a duration of 24 h. Subsequently, the specimens were rinsed twice with 50% ethanol and then transitioned to 70% ethanol for preservation. Ovaries were meticulously dissected from the preserved florets under the aid of a dissecting microscope. To observe the development of the female gametophyte in *G. littoralis*, Whole-mount-stain clearing laser scanning confocal microscopy (WCLSM) methodology was employed [30]. The dissected ovaries underwent a sequential rehydration process involving 50% ethanol, 30% ethanol, and distilled water. They were then mordanted in a 2% aluminum potassium sulfate solution for 20 min, followed by staining with eosin (10 mg/L in a 4% sucrose solution) for a duration of 10–12 h. The stained samples were subsequently subjected to a 20-minute treatment with 2% aluminum potassium sulfate to eliminate the dye from the ovarian walls. Following three rounds of rinsing with distilled water, the samples underwent a stepwise dehydration process involving sequential immersions in 30%, 50%, 70%, 90%, and 100% ethanol for 20 min each. For the purpose of clearing, the dehydrated samples were immersed in an ethanol-methyl salicylate solution (in a volumetric ratio of 1:1) for a period of 2 h, and then submerged in methyl salicylate solution for a minimum of 2 h. The clarified samples were mounted on concavity slides and secured in place with fingernail polish. Subsequently, they were subjected to imaging using a Leica SP8 Laser scanning confocal microscope, following the established protocol [31].

### Suitable habitat prediction analysis based on the maximum-entropy (MaxEnt) model

The geographic distribution information of *G. littoralis* was obtained from the data of China Virtual Herbarium (CVH, https://www.cvh.ac.cn) [32] and Global Biodiversity Information Facility (GBIF, https://www.gbif.org/) [33]. After thorough inspection, cleaning, and de-duplication of all obtained distribution point data, a total of 204 distinct distribution points were collected (Table S1). The ecological factor data used in this study were obtained from the “Traditional Chinese Medicine Resources Spatial Information Network Database” (http://www.tcm-resources.com/), including 55 ecological factors such as precipitation, average temperature, soil type, altitude, vegetation type, etc., which were collected by the Chinese Academy of Traditional Chinese Medicine (CATCM). The database includes 55 ecological factors such as monthly precipitation, monthly temperature, soil type, elevation, topography, vegetation type, etc. The map of China and the administrative divisions of China were obtained from the website of the National Geographic Information Center (http://www.ngcc.cn/ngcc/), at a scale of 1:4,000,000, with the map review number GS(2024)0650, and the coordinate system is WGS 1984.

The collected latitude and longitude data of *G. littoralis* were imported into MaxEnt software, and the ecological factor variables were added at the same time. The parameters were set as follows: 75% of training data, 25% of test data, and the maximum number of iterations was 106. The operation excluded the ecological factors with a contribution rate of 0, and the operation was continued until none of the contribution rates was 0. The accuracy of the operation results was tested by the area under the ROC curve (AUC). The evaluation criteria of AUC are: AUC ≤ 0.6, model prediction failure; 0.6 < AUC ≤ 0.7, model prediction accuracy is poor; 0.7 < AUC ≤ 0.8, model prediction accuracy is average; 0.8 < AUC ≤ 0.9, model prediction accuracy is good; 0.9 < AUC ≤ 1.0, model prediction accuracy is very good, i.e., the higher the value of AUC is, the more reliable the model calculation results are. The results of MaxEnt modeling were imported into ArcGIS software for suitability zoning, superimposed on the map layers, processed the data with normal distribution, and based on its mean, standard deviation and minimum suitability, different suitability zones were delineated: unsuitable habitat, poorly suitable habitat, moderately suitable habitat, and highly suitable habitat.

### Plant materials, genome survey sequencing, genome size and heterozygosity estimation

4–5 slices of fresh young leaf tissues of *G. littoralis* were collected from its wild population from Changjiang Ao Beach, which were sterilized and washed with 70% ethanol, and then the total genomic DNA was extracted by the modified CTAB method [34], and the DNA concentration was detected with a Qubit fluorometer. The integrity and purity of the samples were detected by agarose gel electrophoresis. *Lycopersicon esculentum* (0.88 Gb) was selected as the internal reference, and cell nuclei suspensions of the samples to be tested and the internal reference samples were prepared using a generalized method [35], and further mixed in the appropriate proportions. The stained cell nucleus suspension samples were up-examined using a BD FACScalibur flow cytometer with 488 nm blue light excitation to detect the fluorescence intensity of the emitted light of propidium iodide, and 10,000 particles were collected for each assay. The coefficient of variation CV% was controlled within 5%. Graphical analysis was performed using Modifit3.0 analysis software.

Genome survey sequencing was conducted by Grandomics Biosciences (Wuhan) Co., Ltd. The genome was sequenced using the whole-genome birdshot (WGS) strategy, employing second-generation sequencing technology. According to the characteristics of the genome, a second-generation DNA library of small fragments was constructed, and these fragment libraries were subjected to paired-end sequencing on the MGISEQ2000 platform to obtain whole-genome sequencing data. To ensure the reliability of the reads, the MGI paired-end sequencing raw reads used for the genome survey were first filtered using the fastp (v.0.20.0) to remove low-quality reads, adapters, and reads containing poly-N [36]. To check for contamination, 100,000 reads were randomly selected for comparison with sequences from the NT (Nucleotide Sequence Database) library.

To understand the genomic characteristics of *G. littoralis*, k-mer analysis was performed using Illumina DNA data to estimate genome size and heterozygosity prior to genome assembly. Briefly, 21-mer frequency distributions of quality-filtered reads were analyzed using the Jellyfish program. By analyzing the 21-mer depth distribution of the purified sequencing reads of the 350-bp library in gce and GenomeScope2.0 software [37, 38], we estimated the genome size of *G. littoralis* using the following formula: G = K-num/K-depth (where K-num is the total number of 21 monomers, K-depth denotes the k-monomer depth, and G represents the genome size). The heterozygosity and repeat content of the *G. littoralis* genome were further estimated by combining the results of simulated data with different heterozygosity in Arabidopsis and the frequency peak distribution of 21 kmer.

### Result

#### Umbels morphology at different development stages

**Fig. 1:**
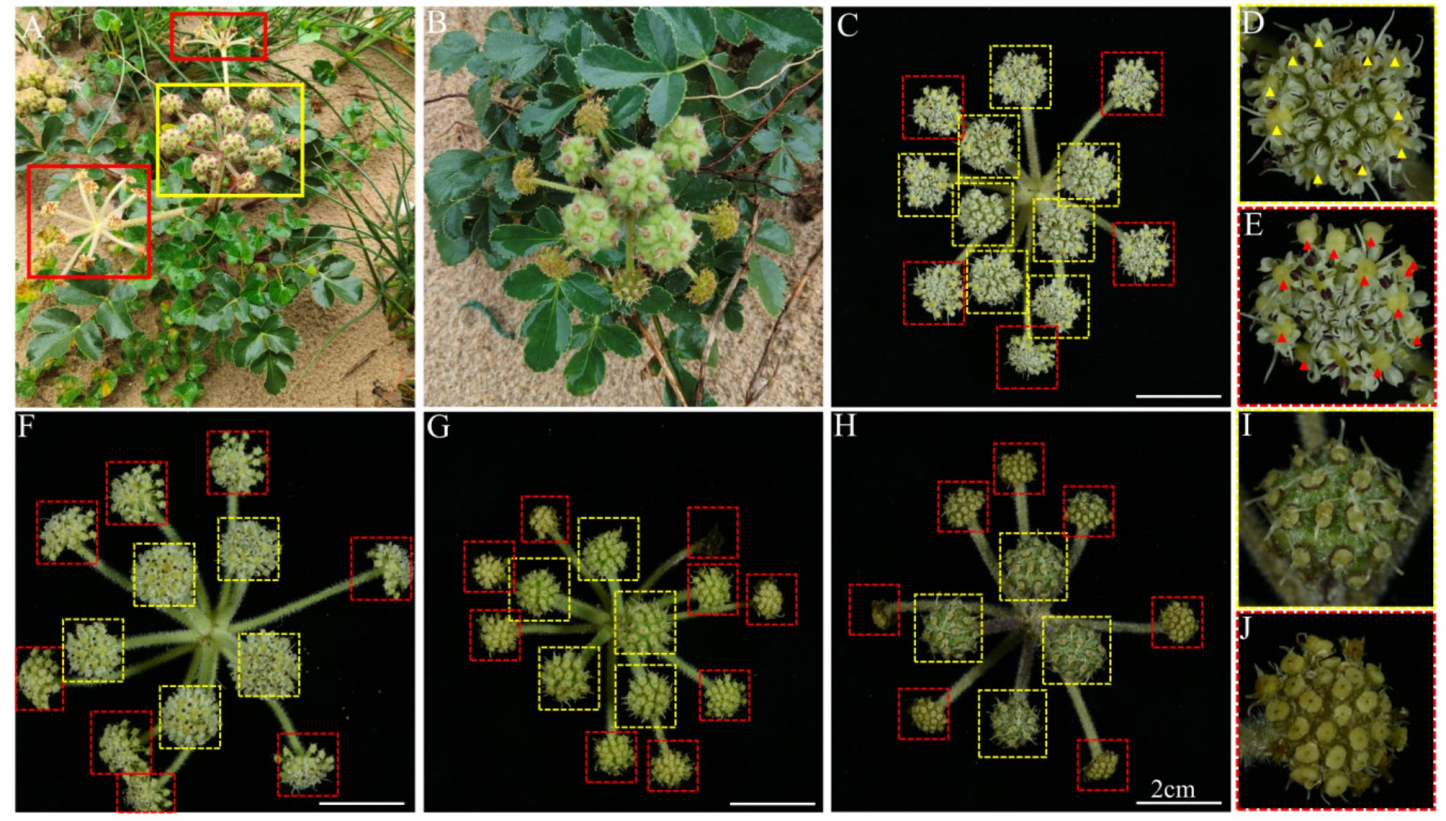
Umbel morphologies during reproductive growth of *G. littoralis*. Yellow and red boxes indicate completely fertile and completely sterile compound umbels, respectively. **A** and **B** show the fruit setting of completely fertile (**A**, yellow box), completely sterile (**A**, red boxes), and partially fertile (**B**) compound umbels in their natural environment. **C**, **F**-**H** show partially fertile compound umbels, with yellow dashed boxes marking fertile umbels and red dashed boxes marking sterile umbels. Yellow arrows in D indicate normally developing stigmas, while red arrows in E show stigmas that have ceased development. I illustrates the completely fertile umbels bearing fruit, with stigmas continuing to extend. J depicts the completely sterile umbels, where fruit development was unsuccessful

As a member of the Apiaceae family, the inflorescence of *G. littoralis* exhibits the characteristics features typical of this family. Observations show that *G. littoralis* has terminal compound umbels, which consist of several umbellules, each containing multiple individual florets. The majority of *G. littoralis* specimens have compound umbels growing from the central part, with lateral umbellules growing from the leaf axils, and a few have lateral compound umbels. In this study, we thoroughly observed and documented the dynamics of *G. littoralis* inflorescence development. Each compound umbel of *G. littoralis* has about 11–20 umbellules. Typically, the central compound umbel has shorter and thicker pedicels, while the peripheral ones are thinner and longer, resulting in a slightly raised center or a flat overall appearance. The compound umbels of *G. littoralis* can be categorized into three types based on their fertility: fully fertile, completely sterile, and partially fertile (containing both fertile and sterile umbellules). Fully fertile compound umbels tend to be terminal (Fig. 1A, yellow box), while fully sterile compound umbels are usually axillary (Fig. 1A, red boxes). The partially fertile umbel bearing fruit in their natural environment is shown in Fig. 1B; this is the most common type in *G. littoralis* populations, and this type of compound umbel also tends to be terminal and typically consists of 3–8 fertile umbels (Fig. 1B, C). The different developmental stages of partially fertile compound umbels from flowering to fruiting are illustrated in Fig. 1C, F-H, where the yellow box indicates fertile umbels and the red box indicates sterile umbels. The number of fertile umbellules correlates with the plant’s size; robust plants tend to have more fertile umbellules, while spindly plants have fewer or none.

In addition, we attempted to discern the differences between the florets in different types of umbels. During the flowering stage in the *G. littoralis* population, we observed two distinct morphologies of florets. Specifically, within the fertile umbels, the stigma elongated from the florets during anthesis, as illustrated in Fig. 1D. In contrast, the florets within the sterile umbels, shown in Fig. 1E, did not exhibit noticeable stigma elongation during anthesis, and their ovaries were relatively small. This led us to suspect that these florets might not be capable of undergoing normal fertilization and fruit development. Consequently, we continued to monitor and observe the respective umbels, which exhibited two distinct fruiting outcomes following the flowering period. As depicted in Fig. 1I, the fertilized ovary exhibited normal enlargement and retained elongated styles, eventually forming mature fruits. Conversely, as shown in Fig. 1J, the styles did not elongate, the ovary did not enlarge, and the entire umbels withered. Based on these observations, we deduced that among the three types of compound umbels in *G. littoralis*, there were two categories of umbels: fertile umbels and sterile umbels, composed of fertile florets and sterile florets, respectively. Florets within the fertile umbels elongate their styles at maturity (Fig. 1D) and continue to elongate during later developmental stages (Fig. 1I), while florets within the sterile umbels do not exhibit style elongation at maturity (Fig. 1E).

**Fig. 2:**
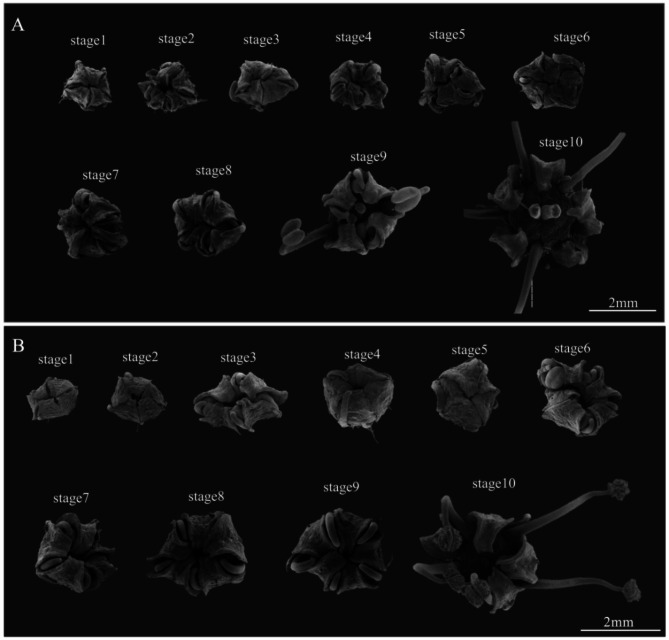
The floral organs of *G. littoralis* at ten developmental stages. (**A**) florets within the fertile umbels, (**B**) florets within the sterile umbels

We therefore conducted separate morphological observations of the florets within the fertile and sterile umbels, categorizing the growth process of the two types of florets into ten stages. In fertile umbels, the ovary and stigma grew and elongated as the florets developed, and the stigma began to grow rapidly between stage 8 and stage 9, with the two stigmas elongating significantly and separating slightly. By stage 9, the plant entered the full bloom stage, with the anthers of florets beginning to rupture and releasing pollen (Fig. 2A, Figure S1). In sterile umbels, although the stamen development appeared normal, the pistil growth stagnated at stage 4 and gradually degenerated in subsequent stages (Fig. 2B, Figure S2), thereby preventing proper pollen reception and resulting in sterility.

### Microsporogenesis and male gametophyte development

**Fig. 3:**
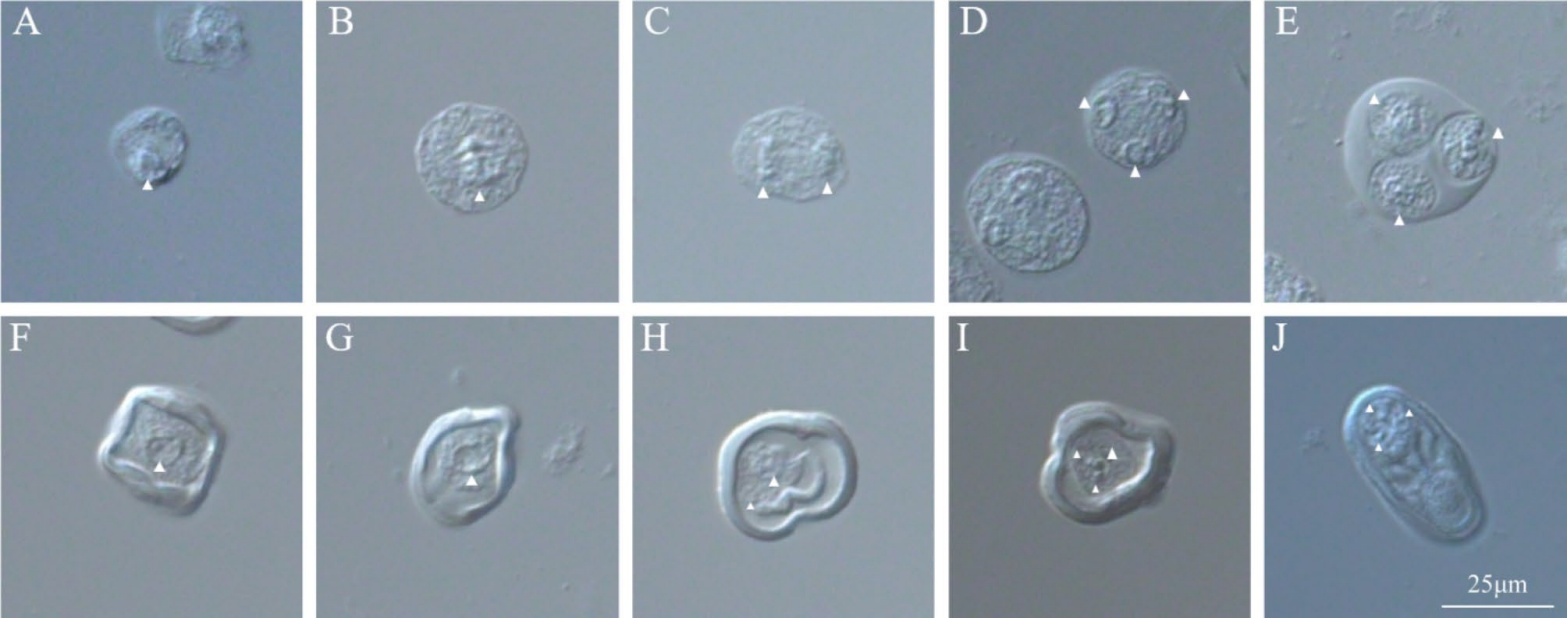
The pollen and male gametophyte development of *G. littoralis* by DIC. (**A**) Pollen mother cell (PMC). (**B**) Pollen metaphase I. (**C**) Pollen anaphase (I) (**D**) Tetrad of four haploid microspores encased in callose, pollen anaphase (II) (**E**) Pollen wall formation and separation of microspores. (**F**, **G**) A single free microspore with central haploid nucleus. (**H**) Two-celled pollen grain, generative cell detached from pollen wall. (**I**) Three-celled pollen grain after second pollen mitosis. (**J**) The mature pollen, note two sperm cells with sperm nuclei

The maturation of male and female gametes is a pivotal phase in the reproductive cycle of plants [39, 40]. Here, we conducted a comprehensive investigation into the pollen and ovule development process of *G. littoralis* and compared it with that of the model plant, *Arabidopsis thaliana*. The pollen development in *G. littoralis* was examined using differential interference contrast (DIC) microscopy, and the developmental stages are illustrated in Fig. 3. The male gametophyte development can be divided into two distinct stages: microsporogenesis and male gametophyte formation. During microsporogenesis, the meiotic events in *G. littoralis* were similar to those observed in dicots, occurring simultaneously. This process commences with the division and subsequent differentiation of germline cells into pollen mother cells (PMCs) (Fig. 3A). The PMCs then undergo their first meiotic division (Fig. 3B, C), followed by meiotic division II (Fig. 3D), leading to the development of tetrad pollen. Cellularization occurs next, forming four individual unicellular haploid microspores (Fig. 3E), marking the initial stage in the male gamete development. Notably, the male gametophyte development in *G. littoralis* shares similarities with *A. thaliana* (Figure S3). As the pollen wall thickens, the mononuclear microspores undergo their first mitotic division, known as pollen mitosis I, resulting in the formation of binucleate pollen (Fig. 3F-H). During this stage, distinct surface features, or bumps, emerge. Binucleate pollen consists of two discernible cells: a larger vegetative cell and a smaller germ cell. Subsequently, the second mitotic division, known as pollen mitosis II, gives rise to trinucleate pollen grains, representing the male reproductive unit. At this point, the nucleus is situated to one side of the pollen grain and comprises one vegetative and two germinal nuclei (Fig. 3I). Ultimately, the developmental process culminates in the formation of mature, ovoid pollen grains, as depicted in Fig. 3J.

### Megasporogenesis and female gametophyte development

**Fig. 4:**
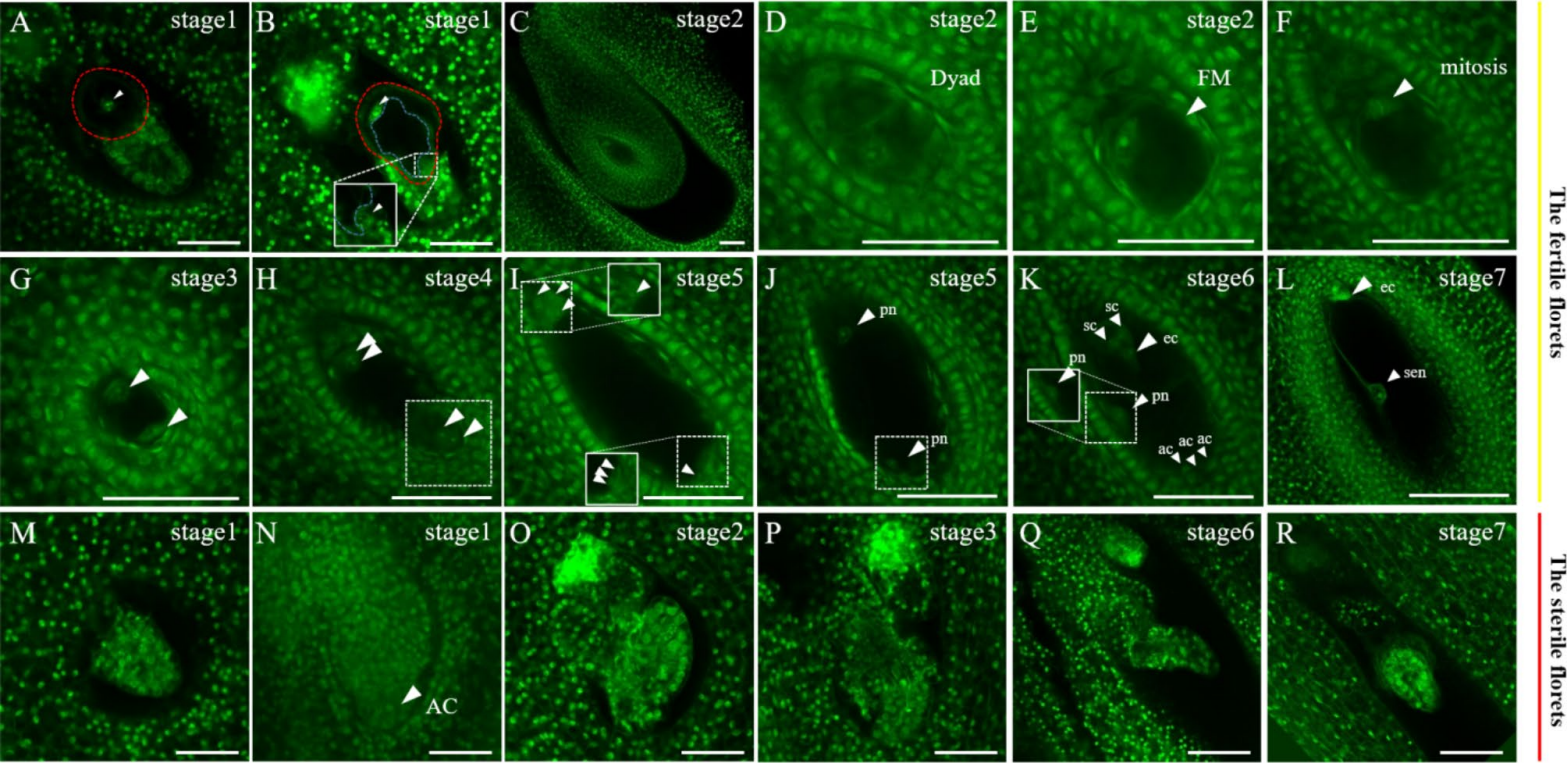
Development of the Female Gametophyte in *G. littoralis.* (**A**, **B**) Specialized cell at the AC stage. (**C**) Morphology of early ovule. (**D**) Dyad: dyad period. (**E**) FM: functional megaspore period. (**F**) First mitosis of female gametogenesis. (**G**) FG3: the two nuclei in the embryo sac gradually moved towards the micropyle end. (**H**) FG4: the embryo sac with four nuclei. (**I**) FG5: embryo sac with eight nuclei. (**J**) FG6: two nuclei moved towards the center without fusion, forming four cell types in the embryo sac, including one egg cell, one central cell, two synergid cells, and three antipodal cells. (**K**) FG7: three synergid cells degenerate at the chalazal end, leaving only one egg cell, one central cell and two synergid cells in the embryo sac. (**L**) Mature embryo sac. (**M**-**R**) Ovule morphology of sterile florets at different developmental stages. Bar = 50 μm

As mentioned above, *G. littoralis* exhibits two types of florets: fertile and sterile (Fig. 1). The anomaly of flowers may also extend to abnormal ovule development. Therefore, we observed the female gametophyte development processes in fertile and sterile florets separately. Referring to the development of the female gametophyte in *A. thaliana* (Figure S4), we delineated the development of the female gametophyte in fertile florets of *G. littoralis* into two stages: megasporogenesis and female gametophyte formation, annotating seven periods of flower development corresponding to female gamete development, as illustrated in Fig. 4C-L. The type of the female gametophyte development of *G. littoralis* is polygonum type, same as *A. thaliana*. Next, the development of *G. littoralis*’ female gametophyte has been described.

The sequential stages of megasporogenesis commence with the archesporial cell (AC), which transforms into the megaspore mother cell (MMC). In the initial archesporial cell stage (Fig. 4A, B), the ovule primordium exhibits protuberances, resulting in the formation of finger-like structures. Subsequently, one of the cells within the central apical tissue undergoes a transformative change in cell fate, transmuting into MMC. Concurrently, the inner and outer integuments fully enclose the embryo sac (Fig. 4C). Meiotic division follows, with the first meiotic division of the MMC resulting in diploid megaspores (Fig. 4D). And then, four haploid megaspores have produced after the meiosis. The chalazal-most megaspore survives and becomes a functional megaspore (FM), and the three micropyle-end megaspores were degenerated. Female gametogenesis then begins, which could be delineated into several stages including FG1-FG7. At the FG1 stage, the female gametophyte is uninucleate, containing a single nucleus within the embryo sac (Fig. 4E). Subsequently, at the FG2 stage, nuclear mitosis, occurs at micropyle-end, leads to the formation of a binucleate embryo sac (Fig. 4F). FG3 is akin to its counterpart in Arabidopsis, with both nuclei translocating to the opposing poles as female gametogenesis unfolds (Fig. 4G). During FG4, a pair of nuclei within the embryo sac concurrently undergo a second mitotic division, culminating in the configuration of an embryo sac harboring four nuclei (Fig. 4H). FG5 witnesses a third mitotic division, engendering eight nuclei within the embryo sac (Fig. 4I). At the FG6 stage, through nuclear migration and cellular differentiation, four distinct cellular types emerge within the embryo sac, comprising one egg cell, two synergid cells, two central cell, and three antipodal cells (Fig. 4J, K). Finally, at the FG7 stage, the three antipodal cells undergo degeneration, one egg cell, one central cell, and two synergid cells be observed in the embryo sac.

In contrast, there is a notable disparity between fertile and sterile florets in the funicle. Ovules within sterile florets cease their development after forming finger-like structures, with no discernible formation of MMC (Fig. 4M-R). Fertile florets, however, feature a substantial specialized cell at the funicle, replete with conspicuous vesicles. Such a structure is conspicuously absent in sterile florets. *G. littoralis* inhabits nutrient-poor coastal sandy soils, environments characterized by severe water stress and insufficient nutrient supply. From an evolutionary perspective, the abortion of some reproductive organs in *G. littoralis* is an environmental feedback mechanism. During flowering, a sufficient number of florets attract pollinating insects, while only a few florets, supported by the substantial specialized cell’s stable water and nutrient supply, can develop normally and produce at least a few viable seeds, thereby maintaining population stability. Thus, we propose that this specialized cell structure, rich in nutrients essential for normal female gametophyte development, is crucial for determining the fertility of these florets.

### Distribution of suitable habitats for *G. littoralis* in China

To predict the suitable habitats for the long-term survival of *G. littoralis* within China’s geographical range, the MaxEnt (Maximum Entropy) model was utilized. This model serves as a valuable tool for delineating fitness zones by integrating multiple parameters, including percentage contribution, replacement importance value, jackknife test, and consideration of environmental limiting factors [41, 42]. For this analysis, the geographic records related to the population of *G. littoralis* (Table S1) were employed to identify the key environmental parameters associated with *G. littoralis* growth and to predict the fitness zones conducive to the persistence of *G. littoralis* within the geographical confines of China.

**Fig. 5:**
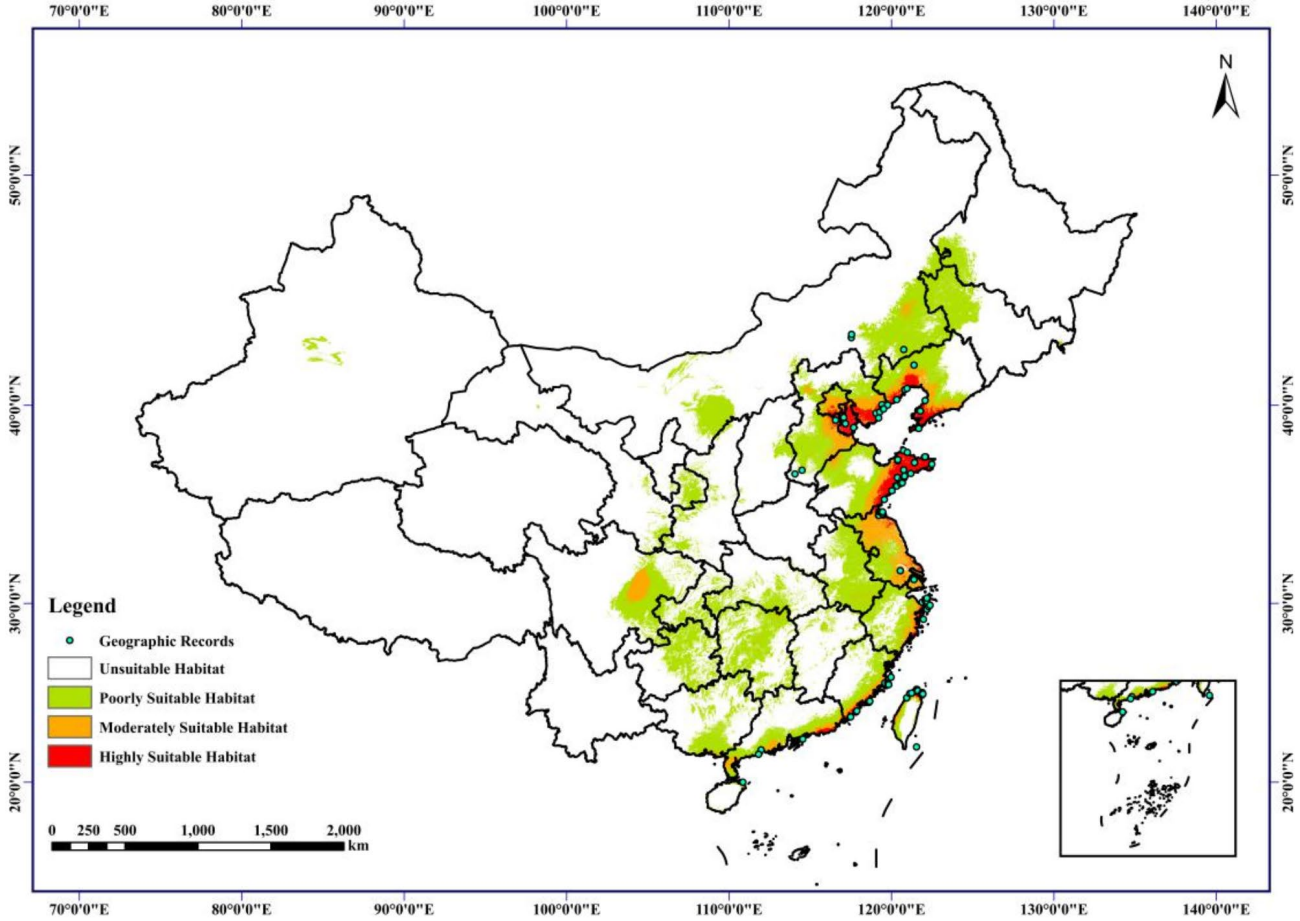
Potential growing distribution of suitable planting regions for *G. littoralis* for different climate change scenarios in China (The map review number: GS (2024)0650). White indicates unsuitable habitat, green: poorly suitable habitat, yellow: moderately suitable habitat, red: highly suitable habitat, black point: distribution record

Based on MaxEnt modeling results, a total of 19 variables were identified as contributing 100% (Table 1), with precipitation of wettest month (bio13), average temperature in August (tmean8), average temperature in September (tmean9), precipitation of driest month (bio14), and the maximum temperature of warmest month (bio5) collectively accounting for over 70% of the contribution. Specifically, the optimal growth of *G. littoralis* occurs under the following climatic conditions: precipitation of wettest month (bio13) ranging from 20 to 800 mm, average temperature in August (tmean8) from 21 to 30 ℃, average temperature in September (tmean9) from 24 to 28 ℃, precipitation of driest month (bio14) from 0 to 180 mm, and the maximum temperature of warmest month (bio5) from 34 to 40 ℃ (Figure S5). Additionally, the Jackknife test revealed that bio2, representing the mean diurnal range, holds significant weight when used independently (Figure S6). These results indicates that temperature and temperature variation are critical factors influencing the suitability of *G. littoralis*. Overall, the favorable growth region for *G. littoralis* in China predominantly resides within the eastern coastal area. The areas with medium to high suitability for *G. littoralis* cultivation are primarily located within the Shandong Peninsula and the Liaoning Peninsula in China, and there are also select regions within inland provinces, including Neimenggu, Sichuan, Hubei, and Henan, where *G. littoralis* cultivation can be successfully undertaken (Fig. 5). These areas are characterized by hilly and plain topography, primarily featuring brown soil with a seasonal frost layer depth ranging from 50 to 100 centimeters. In terms of climate, these regions fall under the category of temperate monsoon climate within the warm temperate zone. The climate exhibits distinctive patterns, characterized by high temperatures and abundant rainfall during the summer, contrasted with cold and dry conditions during the winter months. *G. littoralis* cultivation exhibits distinct temperature requirements throughout various growth and developmental stages. Specifically, seed germination necessitates exposure to low-temperature stratification [43, 44], while the nutritive growth phase demonstrates enhanced progress under higher temperature conditions [45, 46]. The flowering and fruiting stage, on the other hand, demands milder temperature levels [47]. It is noteworthy that these characteristics of *G. littoralis* growth align closely with the prevailing climatic features in the aforementioned regions, rendering them ideal for *G. littoralis* cultivation and seed introduction.

**Table 1:** The contribution of each environmental variable in Maxent modeling

Variable	Description	Percent contribution (%)	Permutation importance (%)
bio13	Precipitation of Wettest Month (mm)	39	6.3
tmean8	Average temperature in August (℃)	13.7	61.2
tmean9	Average temperature in September (℃)	7.1	17.6
bio14	Precipitation of Driest Month (mm)	7	0.3
bio5	Max Temperature of Warmest Month (℃)	6.1	3.4
prec10	Precipitation in October (mm)	6	0.5
bio2	Mean Diurnal Range (Mean of monthly (max temp - min temp)) (℃)	4.8	2.3
bio3	Isothermality	4.5	1.2
bio7	Temperature Annual Range (℃)	2.8	1
slope	slope (°)	2.4	0.6
tmean5	Average temperature in May (℃)	1.9	0.7
trylzjhnl	Cation exchange capacity of soil	1.4	0
tmean4	Average temperature in April (℃)	0.8	2.7
tmean10	Average temperature in October (℃)	0.5	0.9
zblx	Vegetation type	0.5	0
bio15	Precipitation Seasonality (mm)	0.5	0.1
bio17	Precipitation in the driest regions (mm)	0.4	0.4
bio6	The lowest temperature in the coldest month (℃)	0.3	0.4
prec2	Precipitation in February (mm)	0.3	0.3

### Genome size estimation and genome survey of *G. littoralis*

**Fig. 6:**
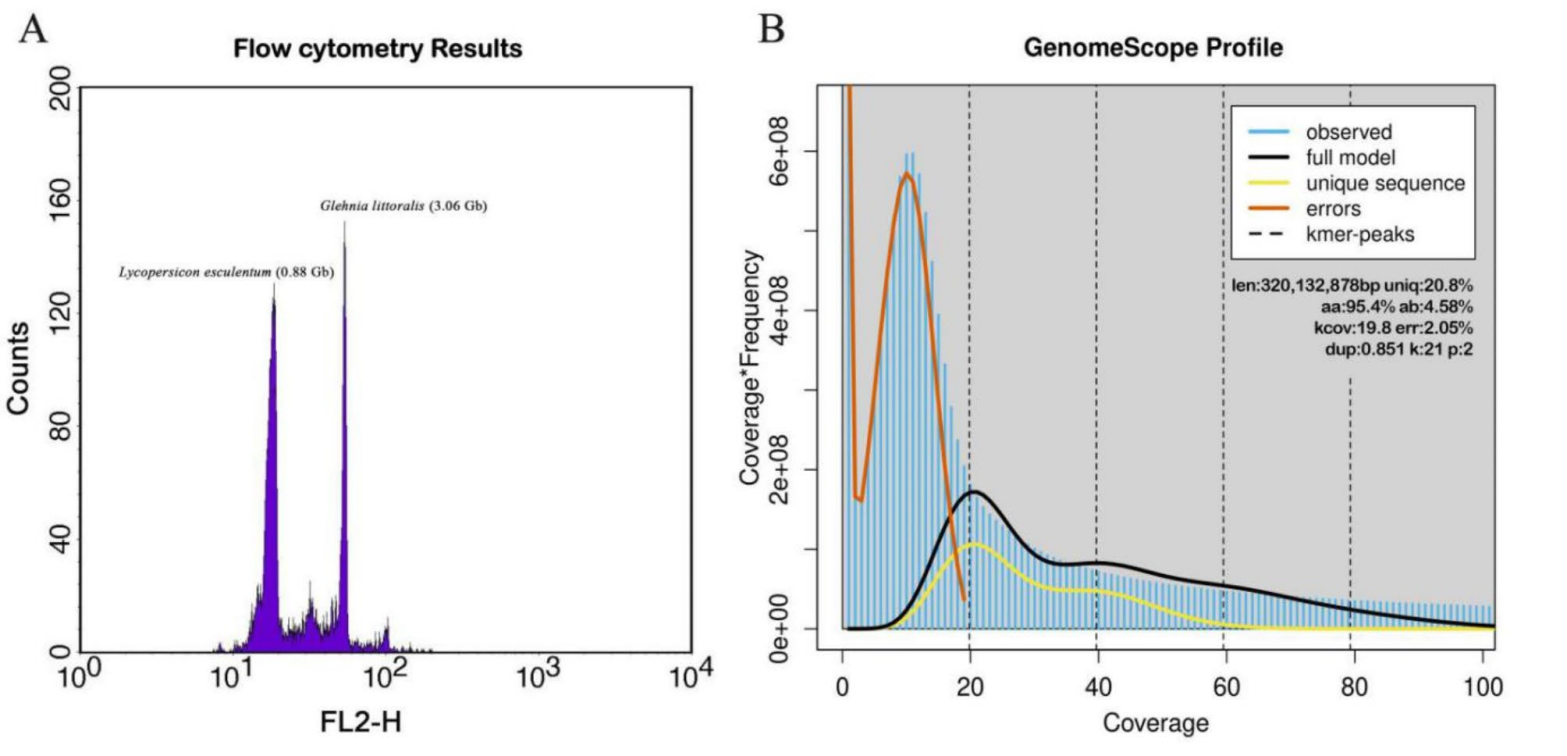
Flow cytometry analysis and Genome survey of *G. littoralis*. (**A**) Histograms showing G0/G1 nuclei peaks of *G. littoralis* compared with *Lycoperscicon esculentum*. (**B**) K-mer (k = 21) distribution calculated by Genomescope. K-mer distribution: blue bars; Modelled distribution without the error K-mers (red line): black line; Maximum K-mer coverage specified in the model: yellow line. len: estimated total genome length from pair end reads; uniq: non-repetitive portion of the genome; kcov: mean K-mer coverage; err: error rate in reads; Dup: duplication rate

High-quality genomes serve as crucial references for conserving plant genetic resources and advancing breeding research, but the essential information for genomic studies of *G. littoralis* remains elusive. To address this gap, we conducted the first genome survey of *G. littoralis* using next-generation sequencing and estimated its genome size through flow cytometry. Utilizing *Lycopersicon esculentum* as an internal reference, we determined the genome size of *G. littoralis* to be 3.06 Gb via flow cytometry (Fig. 6A). The entire genome survey yielded 42.51 Gb of sequence data with an approximate 14.2 × coverage. After quality control, 39.4 Gb of clean data were obtained, with Q20 and Q30 values of 96.42% and 88.30%, respectively (Table S2), indicating the high accuracy of the high-throughput sequencing. Furthermore, the GC content of the reads was approximately 38.50%, suggesting a balanced distribution without significant bias. Subsequently, the entire clean reads were subjected to k-mer analysis to predict the genomic characteristics of *G. littoralis*. Based on the 21-mer frequency distribution, the genome size was estimated to be 3201.32 Mb, basically consistent with the size (3.06 Gb) estimated via flow cytometry. Moreover, the heterozygosity rate and repeat rate were calculated to be 4.58% and 72.9%, respectively (Fig. 6B), indicating that the genome of this species is a relatively complex genome with high levels of heterozygosity and duplication.

## Discussion

The medicinal, nutritional, and ecological significance of *G. littoralis* has prompted heightened interest, leading to extensive research encompassing plant physiology [48], macroscopic morphology [49], seed germination [50], floral organogenesis [51], micropropagation [52], genetics [21, 53], phytochemistry [54], pharmacology [55] and related domains [56]. However, overexploitation and the extensive development of coastal areas have led to a decline in wild populations of *G. littoralis*, which is now listed as a critically endangered plant. Strengthening research on the reproductive biology of *G. littoralis* to elucidate the physiological mechanisms underlying its endangerment, evaluating its suitable growth regions to identify potential areas for in situ and ex situ conservation, and conducting genome sequencing to clarify its genetic background are all critical foundations for the conservation and sustainable utilization of this valuable resource.

As an Apiaceae plant, *G. littoralis* possesses the characteristic inflorescence structure of this family. Based on fertility, the compound umbels of *G. littoralis* can be classified into three types: fully fertile compound umbels, completely sterile compound umbels, and compound umbels containing both fertile and sterile inflorescences. Field observations indicate that the number of fertile inflorescences often correlates with plant size; robust plants have more fertile inflorescences, while weaker plants have fewer or even none. This suggests a close relationship between the fertility of *G. littoralis* flowers and the nutritional status of the plant. Under drought conditions, plant reproductive organs often undergo abortion as a selective evolutionary trait [57]. This partial abortion of reproductive organs allows the plant to complete normal reproductive development despite limited resources [58]. Similarly, competition for assimilates within the plant can lead to reproductive organ abortion [59].

Unveiling the intricacies of gametogenesis in endangered plant species is of paramount significance [60–62], as it provides the foundation for identifying factors contributing to their vulnerability and developing evidence-based conservation strategies. Our observations revealed *G. littoralis* exhibits two types of florets: fertile and sterile (Fig. 1). A notable distinction between these florets is the stigma; the stigma of sterile florets ceases elongation, whereas the stigma of fertile florets elongates and accepts pollen. Additionally, the ovary of sterile florets does not enlarge during flower development (Figure S2), which coincides with abnormal ovule development. In fertile florets, the ovule primordia have a specialized large cell with a giant vacuole at the funiculus, while sterile florets lack this cell and retain a finger-like structure that only slightly elongates during development (Fig. 4). Considering the consistent association between these specialized cells and successful female gametophyte development, as well as the close relationship between reproductive abortion and nutritional status in *G. littoralis*, we hypothesize that the giant vacuole-like structure in fertile flowers may serve as a reservoir of nutrients or hormones essential for normal female gametophyte development. This hypothesis aligns with existing literature on similar structures in other plant species. For example, such vacuole-like structures, referred to as protein storage vacuoles (PSVs) [63], have been documented in various plants. Research indicates that such vacuolar structures arise de novo in the cotyledons of developing peas (*Pisum sativum*) [64], and a similar mechanism may also be operative in *Medicago truncatula* [65]. Plants develop specialized vacuoles in selected tissues that contain substantial quantities of specific materials [66]. For instance, vacuoles in the seed coat’s inner integuments accumulate flavonoids that protect the embryo from detrimental ultraviolet light [67]. In Brassicaceae, vacuoles within myrosin cells along leaf veins store myrosinases (β-thioglucoside glucohydrolases) used for chemical defense against herbivores [68]. Given the harsh and dynamically changing coastal habitat conditions, the formation of this specialized large cell in well-nourished florets may ensure the continuous nutrient supply necessary for normal ovule development, thus supporting reproductive development and population sustainability. Conversely, florets with insufficient nutrient allocation do not form this specialized cell, leading to halted ovule development and less energy cost. However, further research is still needed to provide more direct evidence supporting this hypothesis that these specialized cell structures is vital for determining the fertility of these florets. Meanwhile, our observations of male gametophyte development in *G. littoralis* revealed no significant differences in pollen development between fertile and sterile florets; both types of florets were able to mature normally (Fig. 3). This phenomenon, where sterile florets produce normal pollen, has been reported in many other plants and is thought to help increase pollination chances and promote female reproductive success [69]. *G. littoralis* produces many florets, but under nutrient-limited conditions, excessive flowering and fruiting can result in significant energy depletion. Thus, the additional pollen supply from sterile florets might also contribute to increase pollination chances and promote female reproductive success in *G. littoralis*, serving as an effective strategy for population maintenance under adverse conditions. Collectively, our results, consistent with previous studies, reveal significant reproductive sterility in *G. littoralis*, primarily manifesting as female reproductive abortion. However, our research indicates that fertilization failure in *G. littoralis* is not incidental. We discovered for the first time that a specialized large cell at the funiculus in the ovules of fertile florets may play a crucial role in determining normal ovule development. This finding provides important cytological evidence for the phenomenon of nutrition-limited reproductive sterility in *G. littoralis*.

Our findings suggest that the reproductive capacity of *G. littoralis* is strongly influenced by its surrounding growth environment. Identifying suitable growth areas can provide an optimal environment for *G. littoralis*, offering important guidance for its conservation and efficient cultivation. In this investigation, we conducted the first assessment of the influence of global climate change on the geographical distribution of *G. littoralis* within China. Employing the MaxEnt model, we simulated areas conducive to the growth of *G. littoralis* and identified its primary environmental determinants. We validated our predictions with Receiver Operating Characteristic (ROC) curves, showing a substantial alignment between the predicted suitable growth regions and the actual distribution locales of *G. littoralis*. This validation confirms the precision of our predictive methodology. Our findings also align with similar studies on other plants [70, 71]. Notably, the Area Under the Curve (AUC) values for both the training and test datasets in our study were determined to be 0.981 and 0.984, respectively (Figure S5). These metrics further underscore the utility of our ArcGIS-based MaxEnt modeling approach in delineating suitable cultivation zones for *G. littoralis* [72]. Precipitation and temperature emerge as pivotal environmental determinants governing the distribution of *G. littoralis*. Thus, due to favorable climatic conditions, certain inland regions such as Neimenggu and Sichuan, in addition to the eastern coastal areas, exhibit a high suitability for the cultivation of coastal grass species. This observation provides potential scientific substantiation for the introduction of *G. littoralis* and the enhancement of local agricultural production and industrial diversification. Considering prior research and the current circumstances, it is evident that *G. littoralis* has limited natural distribution points and a small population size. Given the species-specific habitat requirements and the germination kinetics of *G. littoralis* seeds, the effective population size could be further diminished. Populations of wild *G. littoralis* across various habitats currently exhibit levels below the critical threshold essential for their sustained viability, with some populations teetering on the brink of extinction [73]. Consequently, there is an urgent need for research focused on the in-situ and translocation conservation of *G. littoralis*. Our outcomes proffer insights that suggest the establishment of protected areas or reintroduction sites for *G. littoralis* within the projected optimal growth regions. Additionally, future endeavors pertaining to the cultivation of this medicinal plant can be strategically devised, anchored in the key environmental variables elucidated within this study.

Increasing evidence indicates that high-quality genomes can provide essential references for the conservation of plant genetic resources and breeding research [74]. As sequencing technology advances continuously, the application of genome de novo assembly and resequencing techniques facilitates the comprehensive exploration of voluminous genomic data at a cost-effective rate [75]. Indeed, prior to embarking on in-depth whole-genome sequencing, it is imperative to ascertain critical information concerning the degree of heterozygosity, the prevalence of repetitive sequences, GC content, and related characteristics. This knowledge informs the selection of an appropriate assembly strategy for the study being conducted. Using flow cytometry, we estimated the genome size of *G. littoralis* to be approximately 3.06 Gb. Further genome survey analysis revealed that the *G. littoralis* genome is relatively complex, characterized by a high degree of heterozygosity and a high repeat rate. This complexity engenders a propensity for data redundancy during the process of splicing and assembly, making the assembly process challenging. The application of high-accuracy HiFi sequencing technology and Ultra Long technology can effectively enhance the continuity and accuracy of assembling complex genomes like this. In Apiaceae medicinal plants, whole genome sequencing and assembly of Angelica sinensis were performed using HiFi and Hi-C technologies, resulting in a genome size of 2.37 Gb [76]. Additionally, the large and highly heterozygous genome of *Ligusticum chuanxiong* (≈ 3.5 Gb) was successfully mapped to its first Haplotype-phased genome using the aforementioned technologies [77]. In general, the use of these advanced technologies is anticipated to yield a high-quality genome of *G. littoralis*, providing valuable reference data for the conservation of its genetic resources and molecular breeding research.

## Conclusion

*G. littoralis* holds significant medicinal, edible, and ecological value. Conserving and sustainably utilizing this species have emerged as critical focal points due to its endangered status. Here, our study aims to investigate the physiological and ecological mechanisms contributing to its endangerment, as well as to elucidate its genetic background. We first conducted a study on the reproductive development of *G. littoralis*, providing a detailed description of its flowering patterns and floral types. Specifically, fertile and sterile florets constitute fertile and sterile umbels, further forming three compound umbels patterns: completely fertile compound umbels, completely sterile compound umbels, and partially fertile compound umbels Notably, our examination of male and female gametophytes has unveiled a close association between the structural variations in the ovule primordium and the fertility of the florets. Specifically, fertile florets exhibit a specialized giant cell at the base of the ovule primordium, a feature absent in sterile florets. Consequently, this structural attribute likely serves as a significant determinant in the development of *G. littoralis*. Meanwhile, our observation revealed that weaker *G. littoralis* plants produce more sterile florets, while stronger plants produce more fertile florets. Therefore, identifying suitable growth areas is crucial for the conservation and sustainable utilization of *G. littoralis* populations. Our findings reveal that *G. littoralis* thrives in the eastern coastal regions of China, specifically the Shandong Peninsula and Liaodong Peninsula. Furthermore, due to favorable climatic conditions, certain inland regions such as Neimenggu, Sichuan, exhibit a high adaptability to the cultivation of *G. littoralis*. This observation provides a potential scientific basis for the introduction of *G. littoralis*, as well as for increasing local agricultural cultivation and diversification of industries. The conservation of genetic resources of *G. littoralis* is a crucial prerequisite for population sustainability and cultivation utilization. We have provided, for the first time, an assessment of the *G. littoralis* genome size and conducted genome survey analysis, offering essential foundational information for further genome analysis and genetic resource research of *G. littoralis*. Collectively, the observations and outcomes presented herein serve to enhance our comprehension of this coastal plant and offer valuable insights for prospective research endeavors related to the botanical species commonly referred to as *G. littoralis*.

## Electronic supplementary material

Below is the link to the electronic supplementary material.

Supplementary Material 1

Supplementary Material 2

Supplementary Material 3

Supplementary Material 4

Supplementary Material 5

Supplementary Material 6

Supplementary Material 7

Supplementary Material 8

Supplementary Material 9

## Data Availability

The genome survey data was deposited in CNGBdb under the accession number CNP0004903. The direct link for accessing the data is: https://db.cngb.org/search/project/CNP0004903/. The data supporting the findings of this study are available from the corresponding author upon reasonable request.
