# Supplementary figures and images for: Cytological analysis of flower development, insights into suitable growth area and genomic background: implications for Glehnia littoralis conservation and sustainable utilization
Source: BMC Plant Biol. 2024 Sep 30;24:895. doi: 10.1186/s12870-024-05585-5 (PMC11441262; doi:10.1186/s12870-024-05585-5)

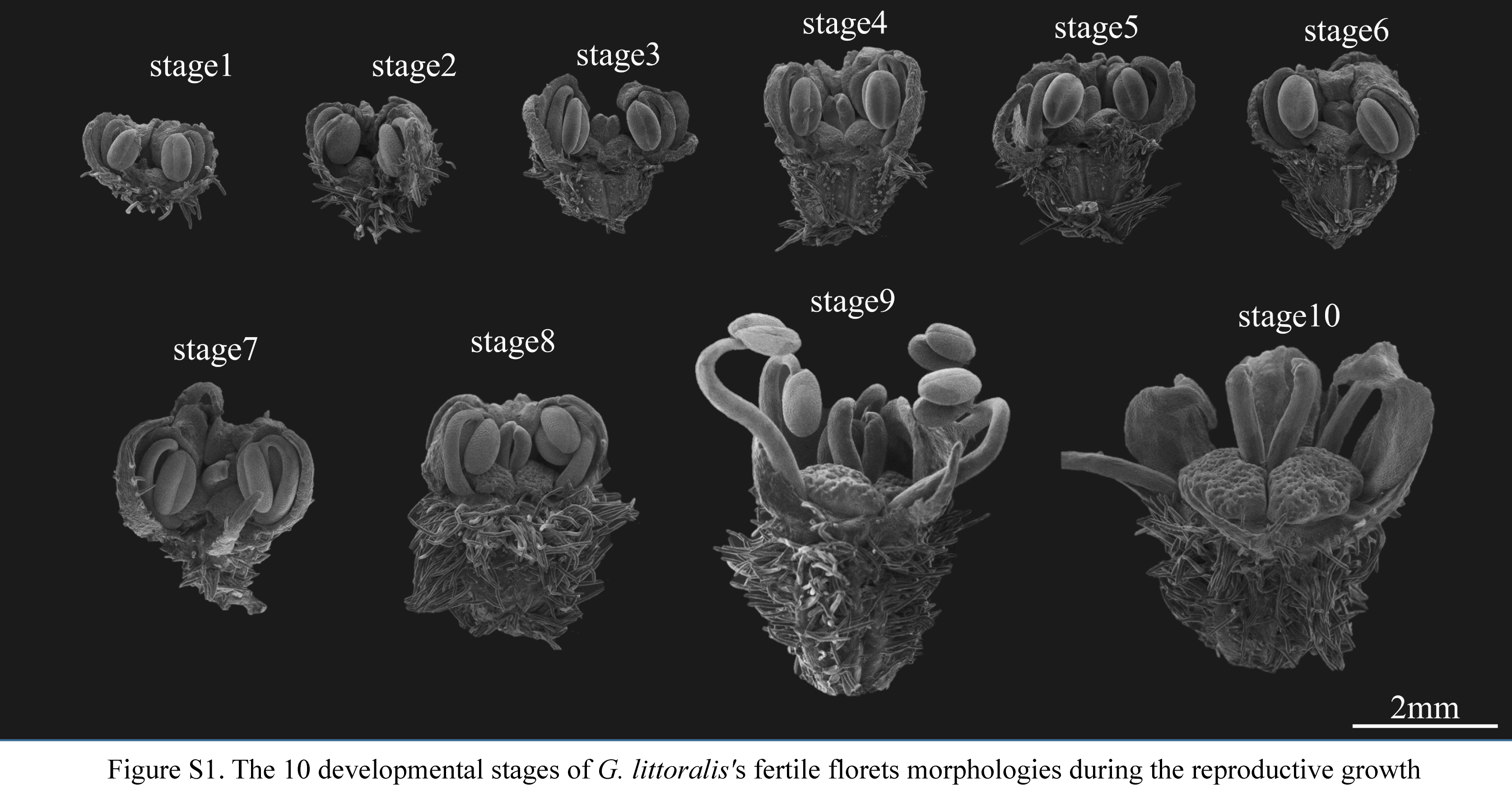

Supplement: Supplementary file 1 — Supplementary Material 1 [file 12870_2024_5585_MOESM1_ESM.png]

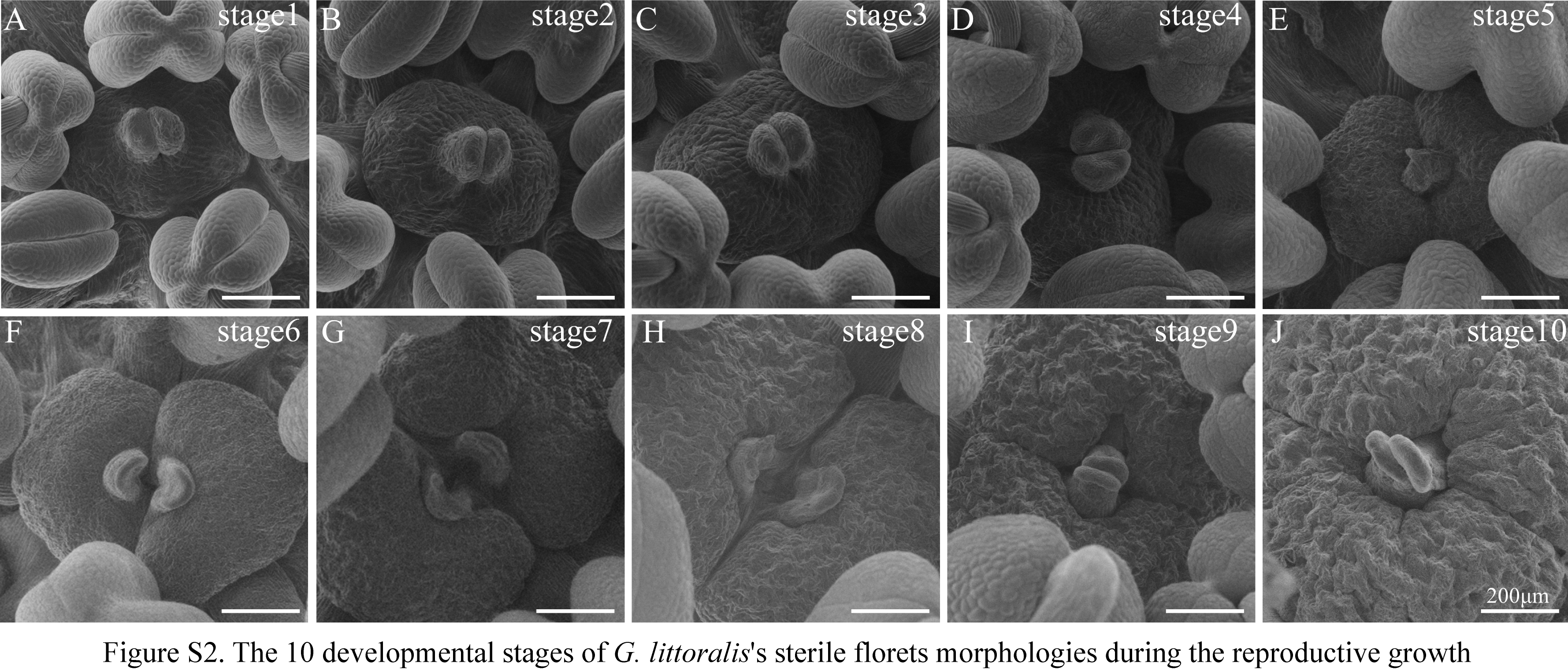

Supplement: Supplementary file 2 — Supplementary Material 2 [file 12870_2024_5585_MOESM2_ESM.png]

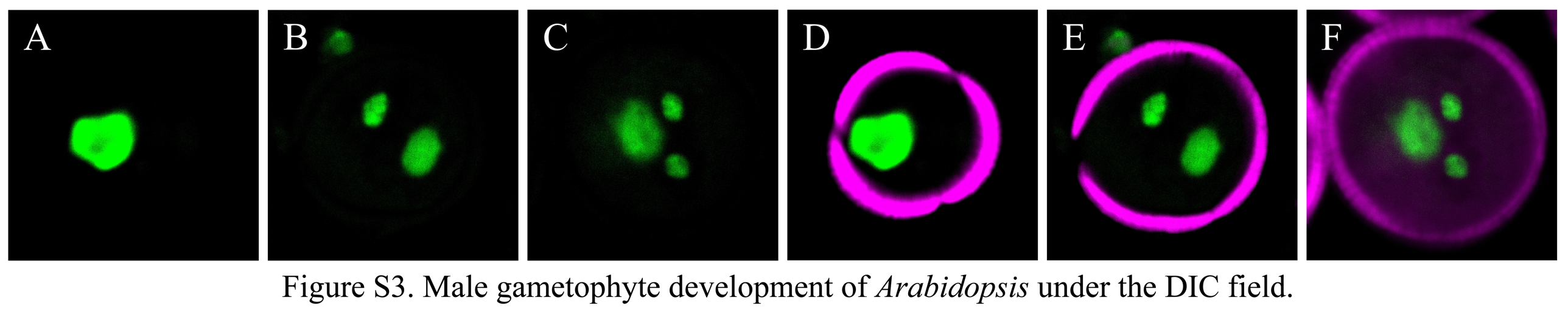

Supplement: Supplementary file 3 — Supplementary Material 3 [file 12870_2024_5585_MOESM3_ESM.png]

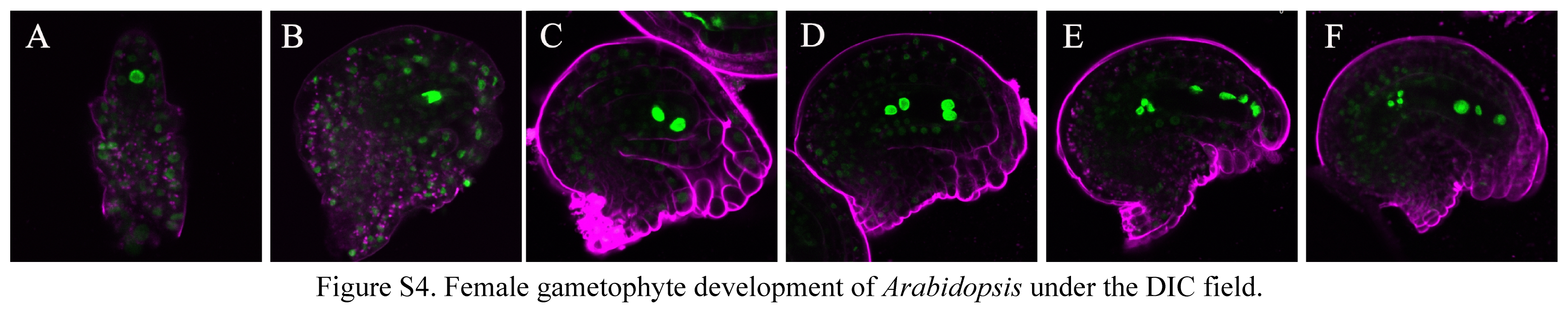

Supplement: Supplementary file 4 — Supplementary Material 4 [file 12870_2024_5585_MOESM4_ESM.png]

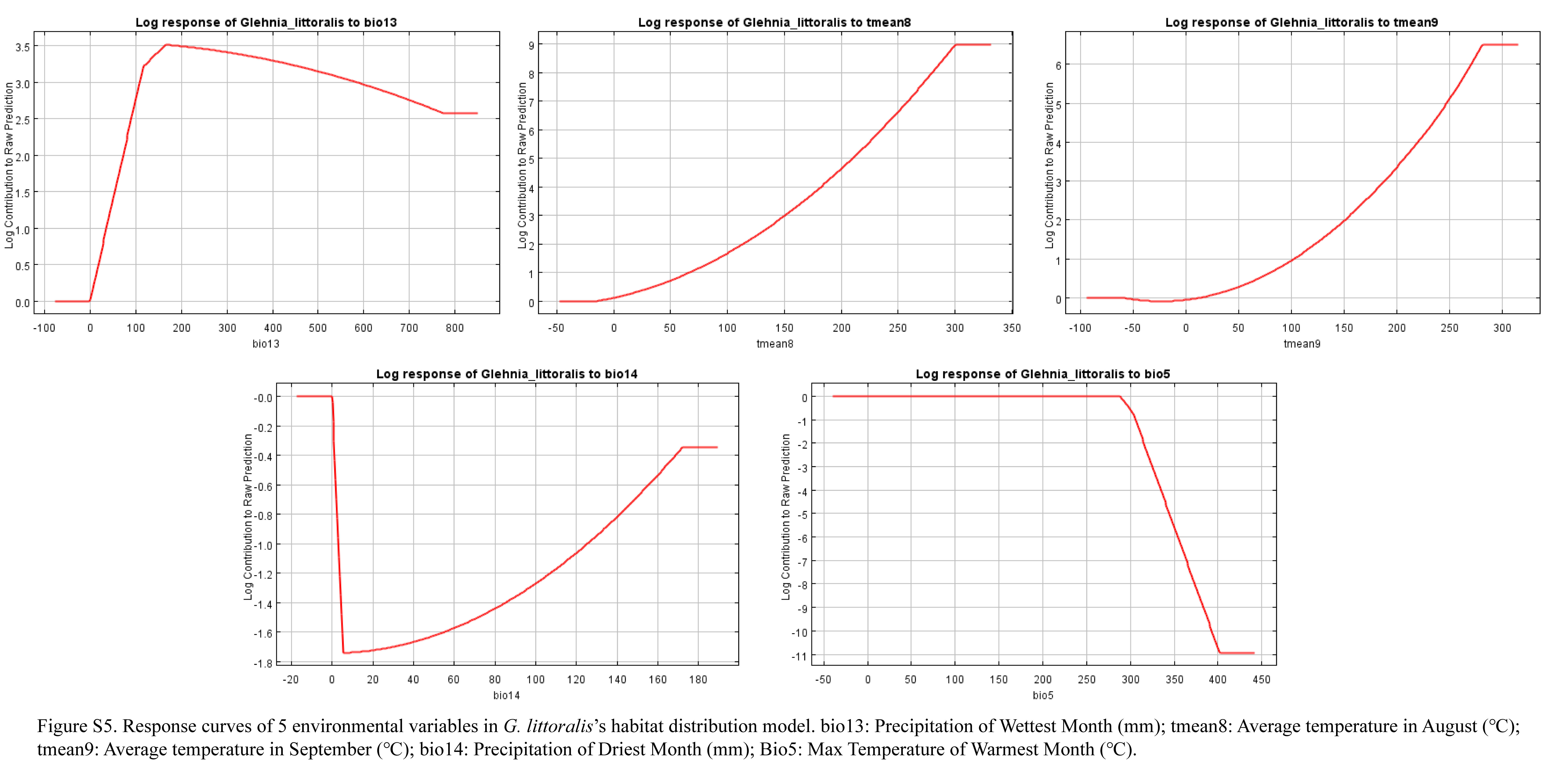

Supplement: Supplementary file 5 — Supplementary Material 5 [file 12870_2024_5585_MOESM5_ESM.png]

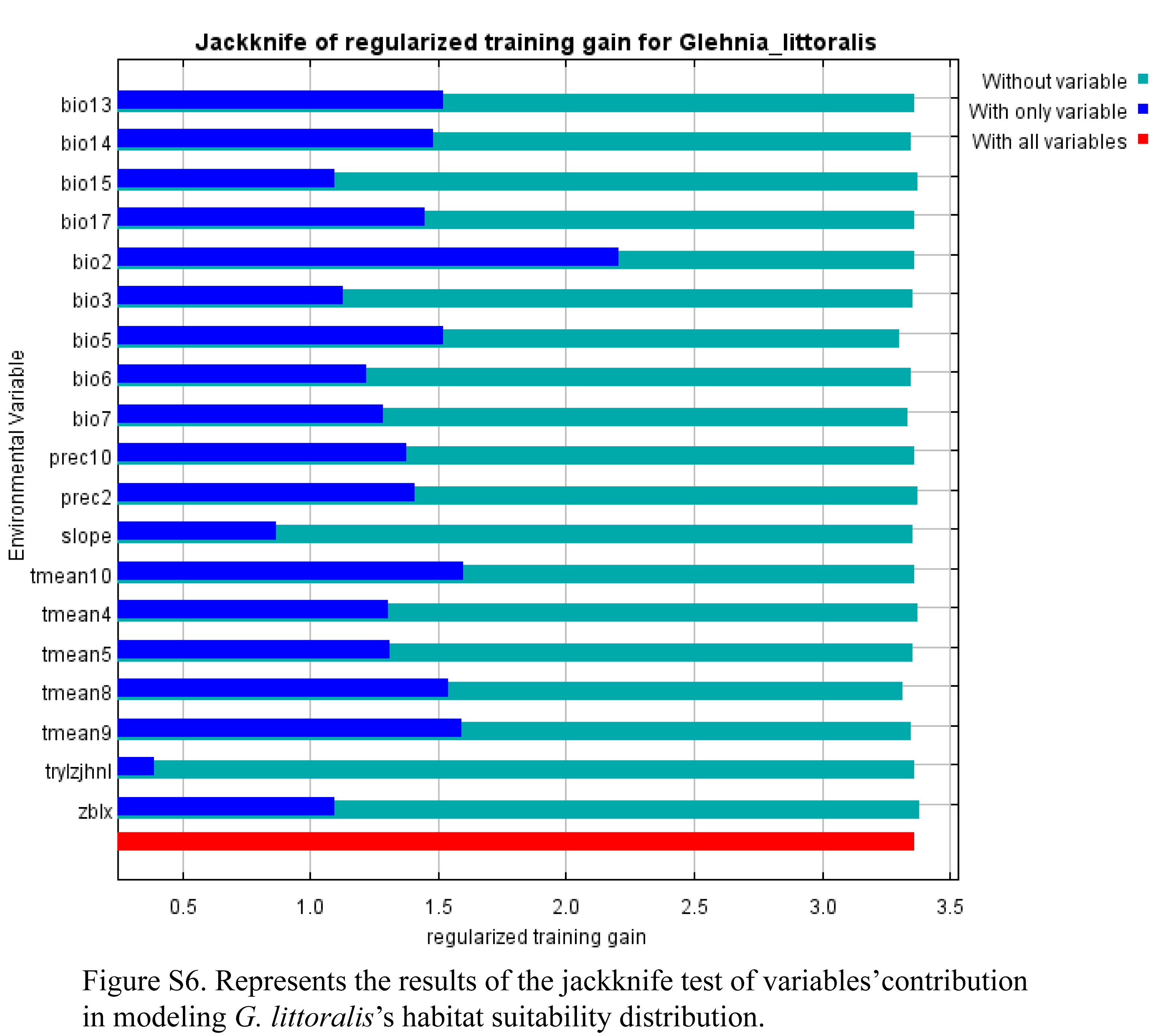

Supplement: Supplementary file 6 — Supplementary Material 6 [file 12870_2024_5585_MOESM6_ESM.png]

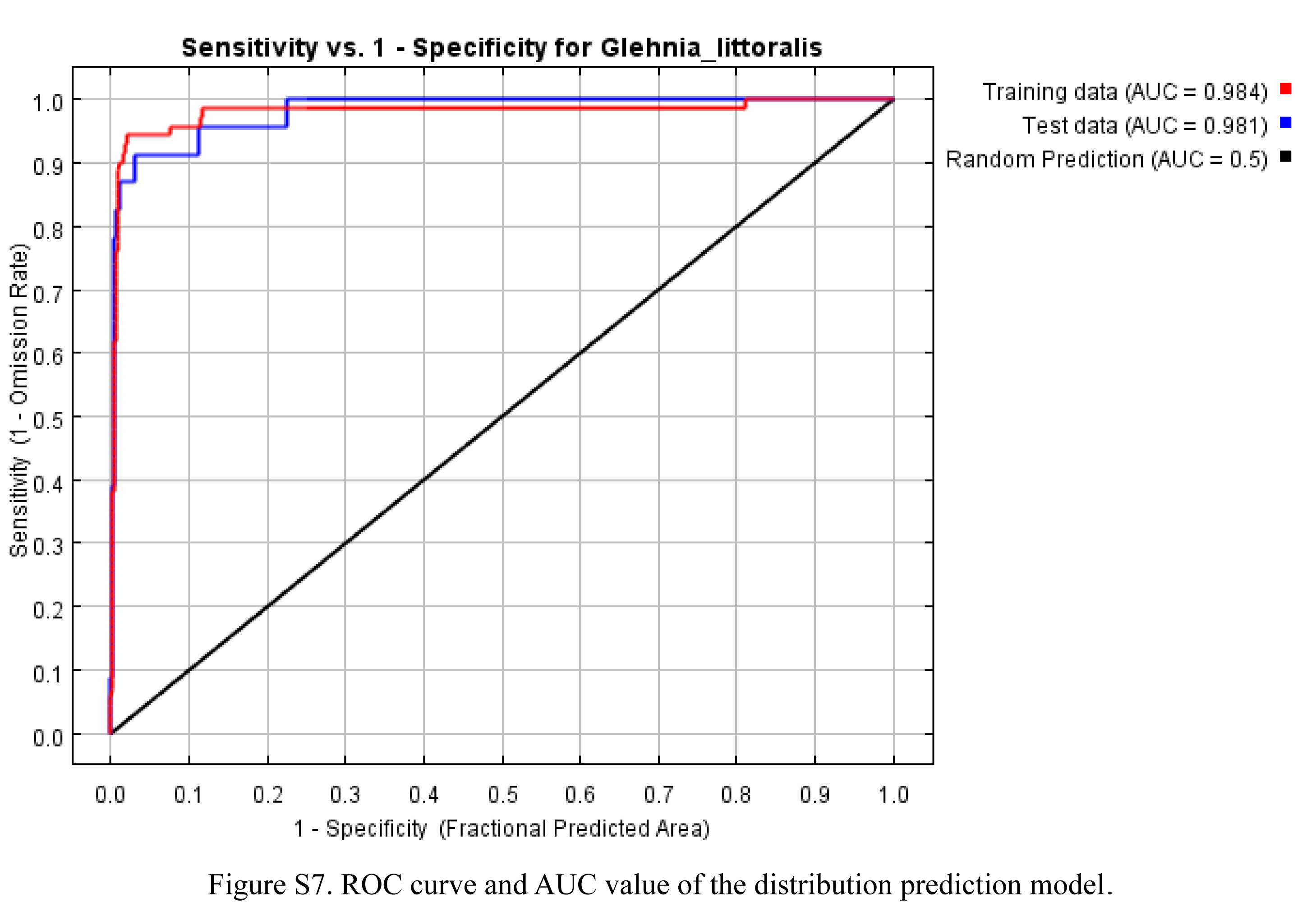

Supplement: Supplementary file 7 — Supplementary Material 7 [file 12870_2024_5585_MOESM7_ESM.png]
